# Pulmonary Oxygen Toxicity Through Exhaled Breath Markers After Hyperbaric Oxygen Treatment Table 6

**DOI:** 10.3389/fphys.2022.899568

**Published:** 2022-05-10

**Authors:** Feiko J. M. de Jong, Thijs T. Wingelaar, Paul Brinkman, Pieter-Jan A. M. van Ooij, Anke-Hilse Maitland-van der Zee, Marcus W. Hollmann, Rob A. van Hulst

**Affiliations:** ^1^ Royal Netherlands Navy Diving and Submarine Medical Centre, Den Helder, Netherlands; ^2^ Department of Anesthesiology, Amsterdam UMC Location AMC, Amsterdam, Netherlands; ^3^ Department of Respiratory Medicine, Amsterdam UMC Location AMC, Amsterdam, Netherlands

**Keywords:** hyperbaric oxygen therapy, hyperoxia, pulmonary oxygen toxicity, exhaled breath markers, volatile organic compounds, treatment table 6, GC-MS, diving and hyperbaric medicine

## Abstract

**Introduction:** The hyperbaric oxygen treatment table 6 (TT6) is widely used to manage dysbaric illnesses in divers and iatrogenic gas emboli in patients after surgery and other interventional procedures. These treatment tables can have adverse effects, such as pulmonary oxygen toxicity (POT). It is caused by reactive oxygen species’ damaging effect in lung tissue and is often experienced after multiple days of therapy. The subclinical pulmonary effects have not been determined. The primary aim of this study was to measure volatile organic compounds (VOCs) in breath, indicative of subclinical POT after a TT6. Since the exposure would be limited, the secondary aim of this study was to determine whether these VOCs decreased to baseline levels within a few hours.

**Methods:** Fourteen healthy, non-smoking volunteers from the Royal Netherlands Navy underwent a TT6 at the Amsterdam University Medical Center—location AMC. Breath samples for GC-MS analysis were collected before the TT6 and 30 min, 2 and 4 h after finishing. The concentrations of ions before and after exposure were compared by Wilcoxon signed-rank tests. The VOCs were identified by comparing the chromatograms with the NIST library. Compound intensities over time were tested using Friedman tests, with Wilcoxon signed-rank tests and Bonferroni corrections used for post hoc analyses.

**Results:** Univariate analyses identified 11 compounds. Five compounds, isoprene, decane, nonane, nonanal and dodecane, showed significant changes after the Friedman test. Isoprene demonstrated a significant increase at 30 min after exposure and a subsequent decrease at 2 h. Other compounds remained constant, but declined significantly 4 h after exposure.

**Discussion and Conclusion:** The identified VOCs consisted mainly of (methyl) alkanes, which may be generated by peroxidation of cell membranes. Other compounds may be linked to inflammatory processes, oxidative stress responses or cellular metabolism. The hypothesis, that exhaled VOCs would increase after hyperbaric exposure as an indicator of subclinical POT, was not fulfilled, except for isoprene. Hence, no evident signs of POT or subclinical pulmonary damage were detected after a TT6. Further studies on individuals recently exposed to pulmonary irritants, such as divers and individuals exposed to other hyperbaric treatment regimens, are needed.

## Introduction

Hyperbaric oxygen therapy (HBOT) is defined by the Undersea and Hyperbaric Medical Society as an intervention in which a patient breathes near 100% oxygen intermittently while inside a hyperbaric chamber at a pressure greater than that at sea level, defined as 1 atmosphere absolute (ATA). For clinical purposes, the pressure in the chamber must be ≥ 1.4 ATA while the subject is breathing near 100% oxygen (Moon, 2019).

Similar to most interventions, HBOT has side effects. Depending on the partial pressure of oxygen and the duration of exposure, neurological and pulmonary symptoms can occur. Neurological symptoms in patients receiving HBOT can include tinnitus, fasciculations, and even seizures, symptoms collectively known as central nervous system oxygen toxicity (CNS-OT) (Wingelaar et al., 2017). Pulmonary side effects of HBOT, known as pulmonary oxygen toxicity (POT), can include retrosternal discomfort, coughing, and lung function impairment, with long exposure leading to lung fibrosis (Smith, 1899; van Ooij et al., 2013).

Clinical signs of CNS-OT and POT resulting from HBOT are very rarely reported, even after patients are exposed to high oxygen pressure for several hours. The treatment table 6 (TT6), a type of hyperbaric therapy, is frequently used to treat divers and caisson workers with decompression illness, resulting from bubble formation by inert gases in tissues due to a decrease in ambient pressure (Brubakk and Neuman, 2003). This treatment regime, originally developed by the US Navy, exposes a patient to HBOT, consisting of oxygen pressure as high as 2.8 ATA, for approximately 5 h (Moon and Mitchell, 2019). In addition to its empirical success in the treatment of diving accidents, such as decompression illness, it is frequently used to manage iatrogenic cerebral gas emboli (Moon, 2019).

The risk of POT can be predicted by measuring the Units of Pulmonary Oxygen Dose (UPTD), an indicator of the reduction in pulmonary vital capacity after exposure to excessive oxygen (Clark and Lambertsen, 1970). Although a very crude instrument, it remains the standard method for predicting pulmonary burden due to hyperbaric oxygen. Pulmonary stress after hyperoxic exposure, however, can be more accurately determined by measuring certain volatile organic compounds (VOCs), such as methyl alkanes in exhaled breath (Wingelaar et al., 2019b).

Early signs of POT after a TT6 remain to be determined. The primary aim of this study was to objectively measure exhaled VOCs indicative of POT after a TT6. In addition, we hypothesized that an increase in these VOCs would be found after a TT6, indicating that subclinical POT would result from hyperoxic exposure. Due to the relatively limited exposure associated with the treatment table, however, we hypothesized that the concentrations of these VOCs would decrease to baseline levels within a few hours after treatment.

## Methods

### Preparation

The study was conducted in accordance with the recommendations of the surgeon general of the Ministry of Defense (reference: DGO/210717019). All subjects provided written informed consent and were free to withdraw from the study at any time. In compliance with national privacy legislation and European Data Protection Regulations (GDPR), no data obtained during the course of this study were included in the participants’ medical files.

All participating subjects were trained hyperbaric nurses and physicians serving in the Royal Netherlands Navy. These subjects underwent HBOT according to schedule in the multiplace hyperbaric chamber (Werkspoor/Boerema) at Amsterdam University Medical Center—location AMC. Although the hyperbaric chamber can accommodate up to 16 individuals, the 14 study subjects were divided into two groups of seven each, with the two groups examined on separate days in compliance with local precautionary policies due to COVID-19.

Subjects were included if they were healthy, non-smoking volunteers, and were medically fit for hyperbaric exposure, in accordance with the Netherlands Ministry of Defense medical fitness requirements for diving, requirements based on the United Kingdom Health and Safety Executive document entitled, “The medical examination and assessment of commercial divers” (HSE MA1), which includes extensive cardiopulmonary function and exercise tolerance tests, as well as neurological examinations (Health and Safety Executive, 2015). Subjects were excluded if they had a recent respiratory tract infection or consumed a moderate amount of alcohol, defined as two or more drinks per day. Use of medications, even over-the-counter supplements, had to be reported, with eligibility for inclusion determined on a case-by-case basis. Prior to the day of the study, alcohol use and strenuous physical training were not allowed because of their possible interference in breath analysis. Hyperbaric exposure was not allowed during the 72 h prior to the trial. Subjects were not allowed to eat or drink for 1 h prior to baseline measurements to avoid contamination of exhaled breath samples. Subjects were provided a uniform diet of bread and marmalade during the day of the test subjects, and the subjects were encouraged to eat, thereby preventing alterations in metabolism due to fasting and accompanying alterations in the molecular composition of the exhaled breath. However, eating was not allowed during the hour before breath sample collection.

### Hyperbaric Exposure

Subjects were exposed to hyperbaric conditions using a US Air Force (USAF) TT6. This table is an adaptation of the original US Navy TT6 and normally used by the Royal Netherlands Navy to treat divers with decompression sickness or arterial gas emboli. During this treatment, the subjects were pressurized to 283 kPa (2.8 ATA; equivalent to a depth of 18 m) for 75 min, with each of the three cycles consisting of breathing 100% oxygen for 20 min and breathing regular chamber air for 5 min. After 75 min, the chamber was slowly depressurized to 192 kPa (1.9 ATA; equivalent to a depth of 9 m), with the subjects breathing oxygen. The treatment at 1.9 ATA consisted of six cycles of alternating 100% oxygen for 20 min and air for 5 min for a total of 2.5 h. The chamber was slowly depressurized to 101 kPa (1 ATA, or surface pressure) with the subjects breathing oxygen. The total test time was approximately 4 h 47 min. Supplementary Appendix S1 shows a graphic representation of the TT6 protocol.

### Collection and Analysis of Breath Samples

Prior to hyperbaric exposure, a baseline exhaled breath sample was collected on-site from each subject for GC-MS analysis (Wingelaar et al., 2019b)*.* Exhaled breath samples were collected 30 min, 2 and 4 h after completing the USAF TT6. Each volunteer was asked to breathe for 5 min through a two-way valve setup with an inspiratory VOC filter (Honeywell, USA) at the entrance aperture to minimize contamination by ambient air pollution. After 5 min, a maximum exhalation was blown into an empty uncoated aluminum balloon (Globos Nordic, Denmark), which had been attached to the exit opening on the valve setup and sealed in place, thereby preventing leakage of ambient air into the balloon. An automatic air pump (Gastec, Japan) was used to pump 500 ml of this exhaled air in 2 min through a steel sample tube with a carbon filter (Tenax GR 60/80, Camsco, USA tube). The tubes were stored in the cold for GC-MS analysis, which was performed after all samples were collected over the two test days.

Four samples of air from the recompression chamber, marmalade jar, oxygen masks and measurement area were also collected to identify possible pollutants or covariables for later identification.

Samples were analyzed by GC-MS (GCMS-QP2010, Shimadzu, Japan), as described in previously published papers on VOC identification after hyperbaric exposures (Wingelaar et al., 2019a, 2020). Briefly, using quickly alternating temperatures and electron ionization, the GC-MS analysis of every breath sample results in a list of ion fragments with their relative intensities per retention time, thereby allowing the construction of raw chromatograms to visualize the data (Honour, 2006; Sánchez-Guijo et al., 2013).

### Statistical Methods and Data Analysis

Analysis of exhaled breath after breathing 100% oxygen for 90 min at a pressure of 253 kPa reported that the emission of various types of alkanes had increased up to 35% (Wingelaar et al., 2019a). Because the hyperoxic exposure in the present study was both longer and of higher pressure, similar or greater increases were expected. Assuming a power of 80% and a significance level of 0.05, a minimum of five subjects would be required to detect such an increase and reject the null hypothesis.

The concentrations of ion fragments before and after TT6 exposure were compared by Wilcoxon signed-rank tests. The ion fragments were subsequently sorted by retention times, and the most distinct chromatograms at each of the breath collection times were used to identify relevant signal peaks. Relevant peaks within each chromatogram were identified based on a signal-to-noise ratio of 100:1.

If a relevant peak on a chromatogram corresponded with an increase in ion fragments, defined as three or more ions, the peaks on the chromatograms were identified by their comparisons with known substances in the National Institute of Standards and Technology (NIST) library.

If multiple substances were eligible for selection due to their having similar or identical percentages, these substances were compared with additional biochemical information, and if applicable, the results of studies of breath analyses and known metabolic associations. Any remaining uncertainty was resolved by discussions among three authors (FJ, TW and PB) until a consensus was reached.

After identification, the signal intensities of these substances were analyzed using the Friedman test, to determine whether the overall changes in signal were significant. Post hoc analysis of samples obtained at the various sampling times was performed using the Wilcoxon signed-rank test with the Bonferroni correction.

All data were compiled and statistical analyses performed in R Statistical Software (v4.0.3; R Core Team 2021), combined with R-packages MBESS (v4.9.0; Kelley 2022), sva (v3.20.0; Leek and Storey 2008), rstatix (v0.7.0; Kassambara 2021). Chromatograms were compared with GCMS Postrun Analysis software (GC-MS Solution version 4.52, Shimadzu Corporation) and substances were identified by using the National Institute of Standards and Technology (NIST) online libraries (NIST Chemistry WebBook, 2021). An alpha of 0.05 was considered statistically significant.

## Results

The fourteen healthy, non-smoking volunteers analyzed in the present study included six men and eight women, of mean age 35.5 ± 6.4 years and mean BMI 24.4 ± 2.9 kg/m^2^. There were no violations of the research protocol before, between and during collection of breath samples.

A total of 60 exhaled breath samples were collected for GC-MS analysis, consisting of 4 samples from each volunteer and four samples of the surrounding air. This resulted in a total of 6543 ion fragments. Of the 56 breath samples, five were excluded due to technical sampling errors. After selecting the significantly differing fragments using a Wilcoxon signed-rank test, 432 ion fragments were eligible for further analysis. After sorting per retention time and applying the 100:1 signal-to-noise ratio, 24 clusters of ion fragments (combined as unknown compounds) required identification. Of these 24 compounds, 11 were identified as known metabolites in humans or were associated with alveolar tissue, pulmonary damage or cell structures, whereas the other 13 substances were of non-human origin, such as food additives, solvents and other pollutants (Hakim et al., 2012). Most of the latter were also detected in the four samples of surrounding air.

**FIGURE 1:**
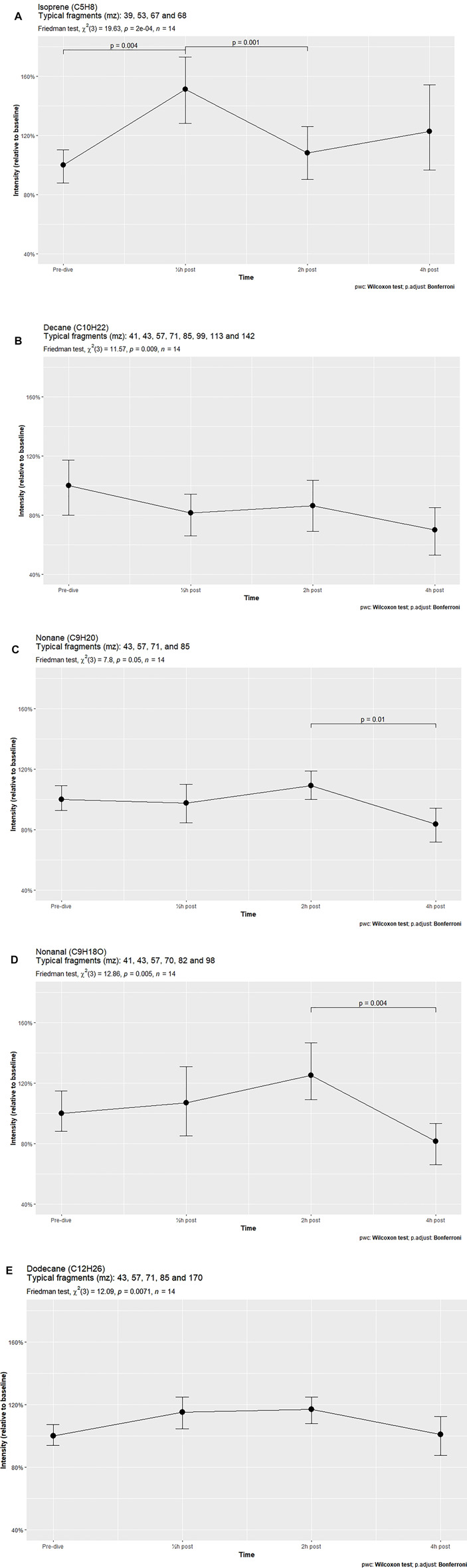
VOCs varying significantly over time in breath samples of subjects exposed to hyperbaric conditions.

Out of the eleven identified human VOCs, five were found to vary significantly in signal intensity. These five VOCs were identified as isoprene, decane, nonane, nonanal and dodecane. Post hoc analysis showed that only isoprene significantly increased 30 min after hyperbaric exposure. The other VOCs demonstrated a fairly constant or decreasing trend, with two VOCs (nonane and nonanal) showing significant reductions in intensity between 2 and 4 h after treatment (Figures 1A–E). The other six identified VOCs did not vary significantly (Supplementary Appendix S2).

## Discussion

The VOC patterns in breath samples of healthy volunteers subjected to hyperbaric conditions were found to vary considerably. Of the eleven identified compounds, five changed significantly over time, with just two of these compounds, isoprene and dodecane, showing a temporary increase in intensity after TT6 exposure. Both increased in intensity 30 min after hyperbaric exposure, but returned to baseline after 2 h or 4 h. In contrast, the other VOCs decreased in signal intensity over time. This pattern was also observed in the compounds that did not vary significantly, with four out of these six compounds showing fairly similar signal intensities over time, comparable to that of decane.

This decreasing overall trend is in accordance with previous studies on POT, which showed lower concentrations of VOCs after repeated exposure to dry hyperbaric oxygen than after “wet/in water” dives in diving simulators or in open water (Wingelaar et al., 2019a). The present molecular analysis showed no signs of POT, and none of the volunteers reported subjective experience of pulmonary symptoms. Thus, the TT6 protocol used in the present study was not associated with a high risk of POT.

None of the subjects experienced any sort of pulmonary symptoms during the day of hyperbaric exposure or on subsequent days. One subject reported waking up the next day with a sensation of a temporary fullness in the middle ear without Eustachian tube dysfunction. This condition, commonly known as “oxygen ear” within the diving community, resolved within a few hours (Shupak et al., 1995).

The hyperbaric treatment table used in the present study is the standard treatment table used at the Royal Netherlands Navy Diving Medical Centre, and is a slightly modified version of the original US Navy TT6. The only difference was the number of air brakes at 9 m, which was three times more frequent in the table used in this study, but without change in the total breathing minutes for both gasses. Specifically, the table used in the present study had three breaks of 5 min each per hour, whereas the US Navy TT6 had one break of 15 min per hour. POT and CNS-OT in rodents may be reduced when a period with greatly increased partial pressure for oxygen is followed by a lower partial oxygen pressure or even a normoxic period at constant ambient pressure (Arieli and Gutterman, 1997; Chavko et al., 2008). This protective effect at similar total oxygen exposure times was greater when alternating hyperoxic-normoxic schedules were more rather than less frequent (Arieli and Gutterman, 1997; Zhang et al., 2019). These findings suggest that the incidence of POT would be lower with our modified TT6, with more frequently alternating oxygen-air periods at 9 m, than with the original US Navy TT6. However, it remains to be determined whether more frequent air brakes affect the development of POT in human subjects.

Similar to the US Navy TT6, the table used in this study was designed to be modified, both by increasing depth and/or prolonging treatment time, depending on subjects’ signs and symptoms and their reaction to treatment (Naval Sea System Command, 2016). These modifications, including additional times of exposure to high oxygen concentrations, are expected to increase VOC production. Thus, the signal intensities observed in this study may be lower than those detected during repeated treatment, or when modifications of the treatment table are used for a severely ill patient.

### Identified Compounds and Their Intensity Patterns

As in earlier studies on POT, most of the identified VOCs were alkanes and methyl alkanes. These compounds, especially the non-branched or straight chain alkanes such as nonane or decane, are believed to be products of oxidative stress, resulting from the action of reactive oxygen species (ROS) on polyunsaturated fatty acids in the lipid bilayers of cell membranes. This process, called lipid peroxidation, disrupts the cellular membrane and can lead to cell apoptosis (Ayala et al., 2014). The origin of the methyl alkanes, such as 3-methylnonane and 3-methylheptane, has not been determined. Studies have suggested that these compounds originate from the same lipid peroxidation process as straight chain alkanes, whereas other studies have suggested that these compounds have different origins (Kneepkens et al., 1994; Phillips et al., 2003). Substances not originating from lipid peroxidation, such as nonanal, methylcyclohexane and isoprene, have been associated with inflammatory processes, oxidative stress responses and cellular metabolism (Gaida et al., 2016; Lamote et al., 2017; Wang et al., 2018).

Regardless of their origin, all identified alkanes have been associated with pulmonary diseases, or with irritants and ROS-inducing processes such as asthma, COPD, air pollution, smoking habits, hypoxia and various types of lung cancer (Filipiak et al., 2012; Harshman et al., 2015; Gaida et al., 2016; Blanchet et al., 2017).

Most of these alkanes, such as 3-methylheptane or decane, showed strikingly similar intensity patterns at all post-exposure times. For example, short-chain alkane intensities were lower 30 min after exposure than at baseline, increasing slightly at 2 h and decreasing further at 4 h. Only the longer and heavier alkanes such as dodecane (C12H26) and tetradecane (C14H30) demonstrated parabolic-shaped intensity graphs, with increases at 30 min and 2 h, followed by a reduction to baseline levels at 4 h.

Although lipid peroxidation may explain the increased intensities of some alkane and methyl alkane compounds after hyperoxic exposure, the reasons underlying the decreased intensities of other VOCs remain unclear. Various hypotheses have been suggested, including the temporary upregulation of antioxidant mechanisms, such as increases in ROS-scavenger enzyme activity, increased (methyl) alkane metabolism and other as yet unknown processes (Pietarinen-Runtti, 2000). Several studies on VOCs in cancer patients have found reduced levels of alkanes and methylalkanes in breath samples. These differences may be due to metabolic alterations resulting from the illness itself or increases in cytochrome P450 activity and other metabolic processes that convert alkanes to alcohols, which are highly soluble in blood and tissues and therefore not as volatile as less soluble compounds (Hakim et al., 2012; Blanchet et al., 2017).

In agreement with previous findings on hyperbaric-hyperoxic exposures, methylcyclohexane was detected in the present subjects (van Ooij et al., 2014; Wingelaar et al., 2019a). This compound is frequently detected in VOC analysis of subjects with smoking habits, benign lung diseases and malignancies such as colorectal cancer (Altomare et al., 2015; Wang et al., 2018).

Isoprene, a lipophilic hydrocarbon with a low boiling point (34°C), is frequently detected at high concentrations in human breath samples. This compound is part of the cholesterol synthesis pathway, with its concentration in exhaled air being highly variable. Because isoprene in breath is little affected by exogenous exposure, such as uptake through food, and is not produced in the respiratory tract, it can be used to monitor cellular metabolic processes. Due to its lipophilic properties and low boiling point it is also an indicator of ventilation rate. Exhaled isoprene levels increase markedly at the start of physical exercise when ventilation rate and metabolism increase, and decrease during sleep when metabolism and ventilation are reduced (King et al., 2009). Although isoprene is an antioxidant in plants (Pollastri et al., 2021), it has not been shown to have antioxidant activity in humans. The present study found that isoprene levels were significantly higher 30 min after exposure than at baseline, but decreased at 2 h. Generally, exhaled isoprene levels tend to rise in the morning and peak around noon, dip in the late afternoon, and level out in the evening, with this pattern being more distinctive in patients with asthma than in healthy individuals (Wilkinson et al., 2019). The individuals in the present study showed the same pattern, suggesting that they had experienced minor pulmonary irritation or that the pattern was due to increased mobilization of the test subjects after they had been seated for 5 h.

### Strength and Weaknesses

To our knowledge, this study is the first to quantify and identify VOCs after a hyperoxic hyperbaric exposure using a TT6. Typically, the occupational diving population is male-dominated. The present study, however, included six men and eight women, making these findings useful for recreational divers as well.

The present study had several limitations. First, the subjects of this study were fit, healthy and relatively young military hyperbaric personnel. Prior to this study, they had no recent pulmonary exposure to known irritants, such as hyperbaric exposure in the days prior to the trial, prolonged exposure to normobaric oxygen or tobacco smoke or recent respiratory tract infections. Findings in these study subjects may differ from those in stricken divers requiring recompression therapy after diving accidents. Hyperbaric treatment of injured divers and patients with prior exposure to increased partial oxygen pressure may result in additional hyperoxic stresses, leading to increased VOC intensities and the development of POT. Nevertheless, like all therapeutic interventions, the benefits of treatment should be weighed against its potential disadvantages. Refraining from necessary hyperbaric therapy due to fear of inducing POT is most likely to disadvantage patients. In the absence of overwhelming indications of POT, patients should receive hyperbaric therapy.

The identification of VOCs remains a complex process; especially if the software identifies multiple possibilities. Therefore, the molecular origin of some of the ion clusters selected by GC-MS could not be determined with absolute certainty. For example, one of the ion clusters showed 98% similarities with octane, nonane and 2,4-dimethylheptane. Although this indicates that this cluster very likely matches one of these compounds, the exact compound could not be determined. Isomers, such as nonane and 2,4 dimethylheptane, frequently show similar findings, making them hard to distinguish by GC-MS analysis. All three compounds are of human origin, have been found in breath samples of patients affected by asthma or various malignancies, and could be fragments of disrupted cellular membranes (Haick et al., 2014;The Human Metabolome Database, 2021). However, because this study sought to objectify POT by measuring changes in VOCs, the exact identification of detected compounds was regarded as less important and can be determined in future studies.

By design, there was no control group in this study. Creating a blinded control group for a hyperbaric study is possible, but has its limitations (Lansdorp and van Hulst, 2018). For example, the subjects of this study, all of whom were experienced hyperbaric personnel, would likely know if they were exposed to the regular TT6 pressures or to a sham regimen. Furthermore, breathing ambient air with 21% oxygen instead of 100% at depth exposes the lungs to a higher partial oxygen pressure than normobaric normoxic exposure. Thus, the control group would still be exposed to the irritant, albeit to a lesser extent. Additionally, breathing air or other gas mixtures with inert gas fractions such as nitrox or heliox for a prolonged amount of time can cause small venous gas emboli, which are filtered out in by the lungs and exhaled, causing intravascular abrasion and endothelial injury (Arieli, 2017; Yu et al., 2017; Hess et al., 2021). These endothelial microinjuries activate various inflammatory responses that could alter the VOC pattern in breath samples, thus obscuring the effects on POT (Mckillop, 2011).

## Conclusion

Eleven VOCs were identified in breath samples of subjects exposed to the TT6. Five of these VOCs demonstrated significantly varying signal intensities over time, but only one compound, isoprene, displayed a clear increase in intensity right after hyperbaric exposure. Although our study included healthy military personnel, not patients or stricken divers, there was no indication found that the TT6 induced significant POT or caused detectable subclinical pulmonary damage. These findings indicate that the therapeutic benefit of this treatment table outweighs the potential harm from POT, showing that the TT6 is safe for clinical use. Studies of the pulmonary burden of a TT6 on patients recently exposed to pulmonary irritants, including stricken divers, as well as other hyperbaric treatment regimens are needed to further assess POT after hyperbaric hyperoxic treatments.

## Data Availability

The datasets presented in this article will be made available upon reasonable request. Requests to access the datasets should be directed to fjm.d.jong@mindef.nl.

## Supplementary Material

Supplementary Figure 1 Schematic showing of the recompression table (USAF TT6) used in this study. In contrast to the USN TT6, in which subjects are exposed to 100% oxygen for 60 min and ambient air for 15 min at a recompression depth of 9 meters, the TT6 in the present study exposed subjects to six cycles of 100% oxygen for 20 min and ambient air for 5 min at a recompression depth of 9 meters. (Source: Zuurstofbehandelingstabel 6, p.19, Instructie Werken onder Overdruk – 003, Ministerie van Defensie 2019).

Supplementary Figure 2 VOCs not varying significantly over time in breath samples of subjects exposed to hyperbaric conditions.
