# Supplementary material for: Pulmonary Oxygen Toxicity Through Exhaled Breath Markers After Hyperbaric Oxygen Treatment Table 6
Source: Front Physiol. 2022 May 10;13:899568. doi: 10.3389/fphys.2022.899568 (PMC9127798; doi:10.3389/fphys.2022.899568)
Supplement: Supplementary file 1 [file DataSheet2.pdf]

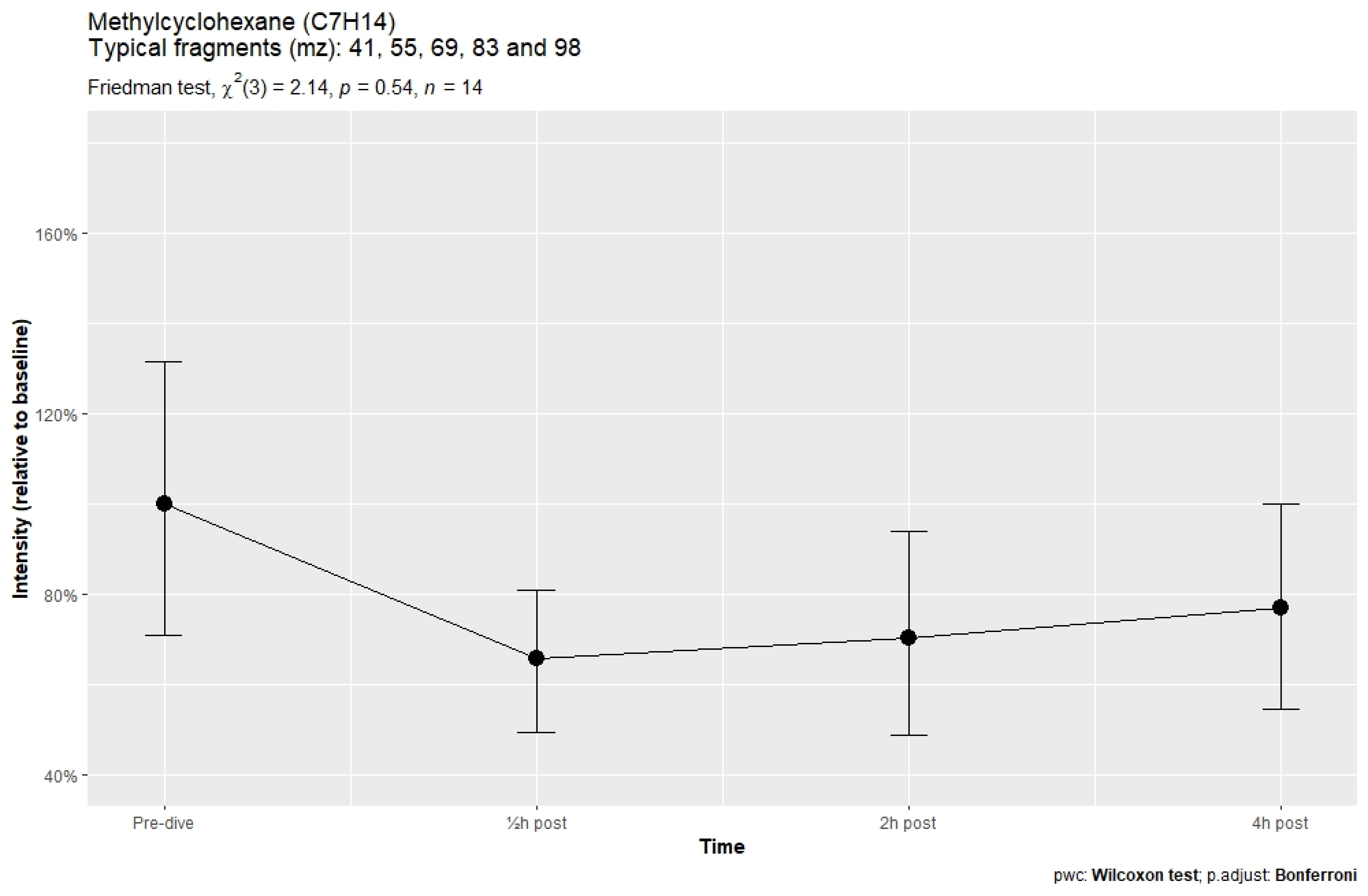

Methylcyclohexane (C7H14)
Typical fragments (mz): 41, 55, 69, 83 and 98
Friedman test, $\chi^2(3) = 2.14$, $p = 0.54$, $n = 14$
160%
120%
80%
40%
Intensity (relative to baseline)
Pre-dive
½h post
2h post
4h post
Time
pwc: **Wilcoxon test**; p.adjust: **Bonferroni**

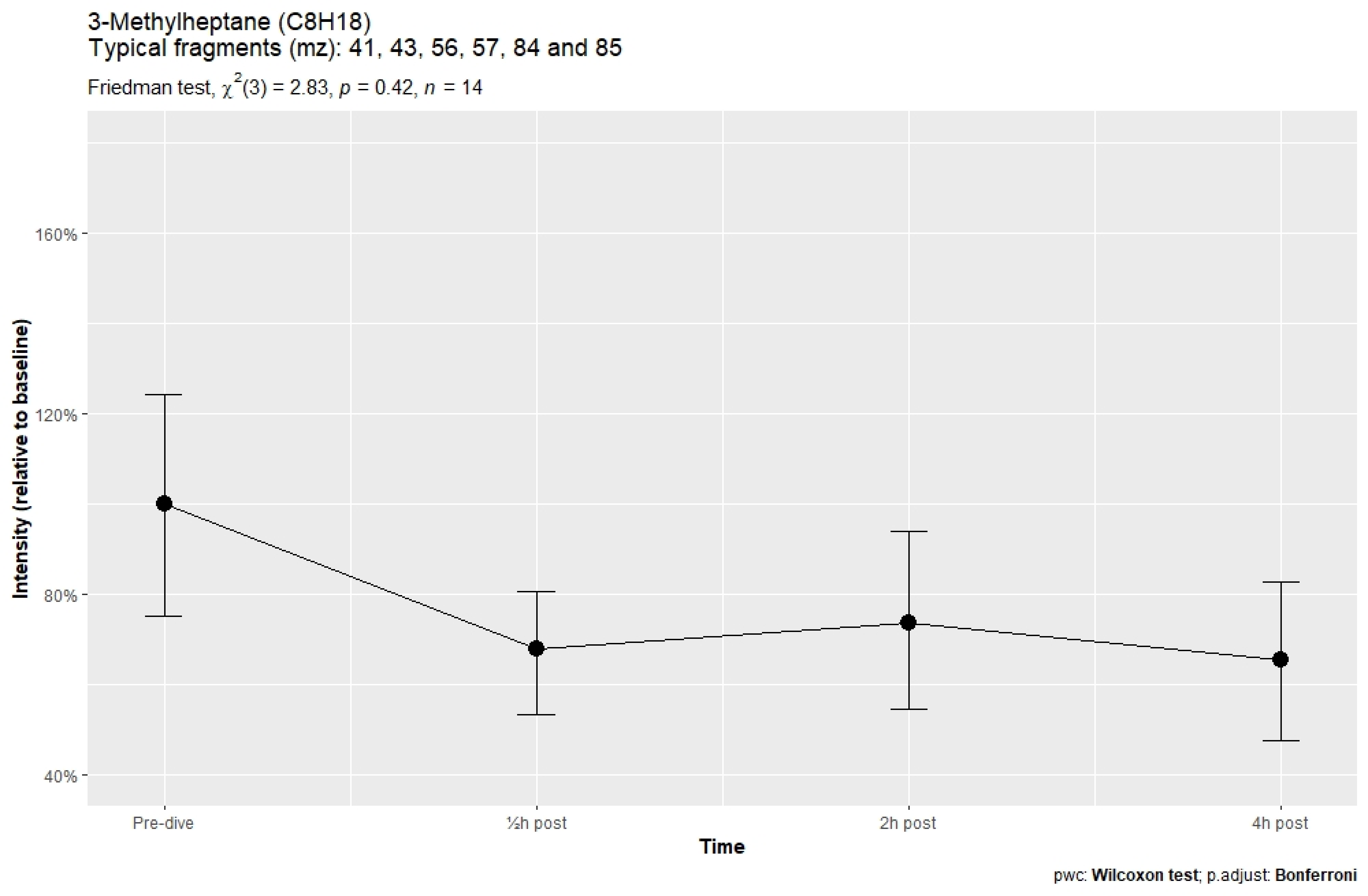
3-Methylheptane (C8H18)
Typical fragments (mz): 41, 43, 56, 57, 84 and 85
Friedman test, χ2(3) = 2.83, p = 0.42, n = 14
Intensity (relative to baseline)
160%
120%
80%
40%
Pre-dive
½h post
2h post
4h post
Time
pwc: Wilcoxon test; p.adjust: Bonferroni

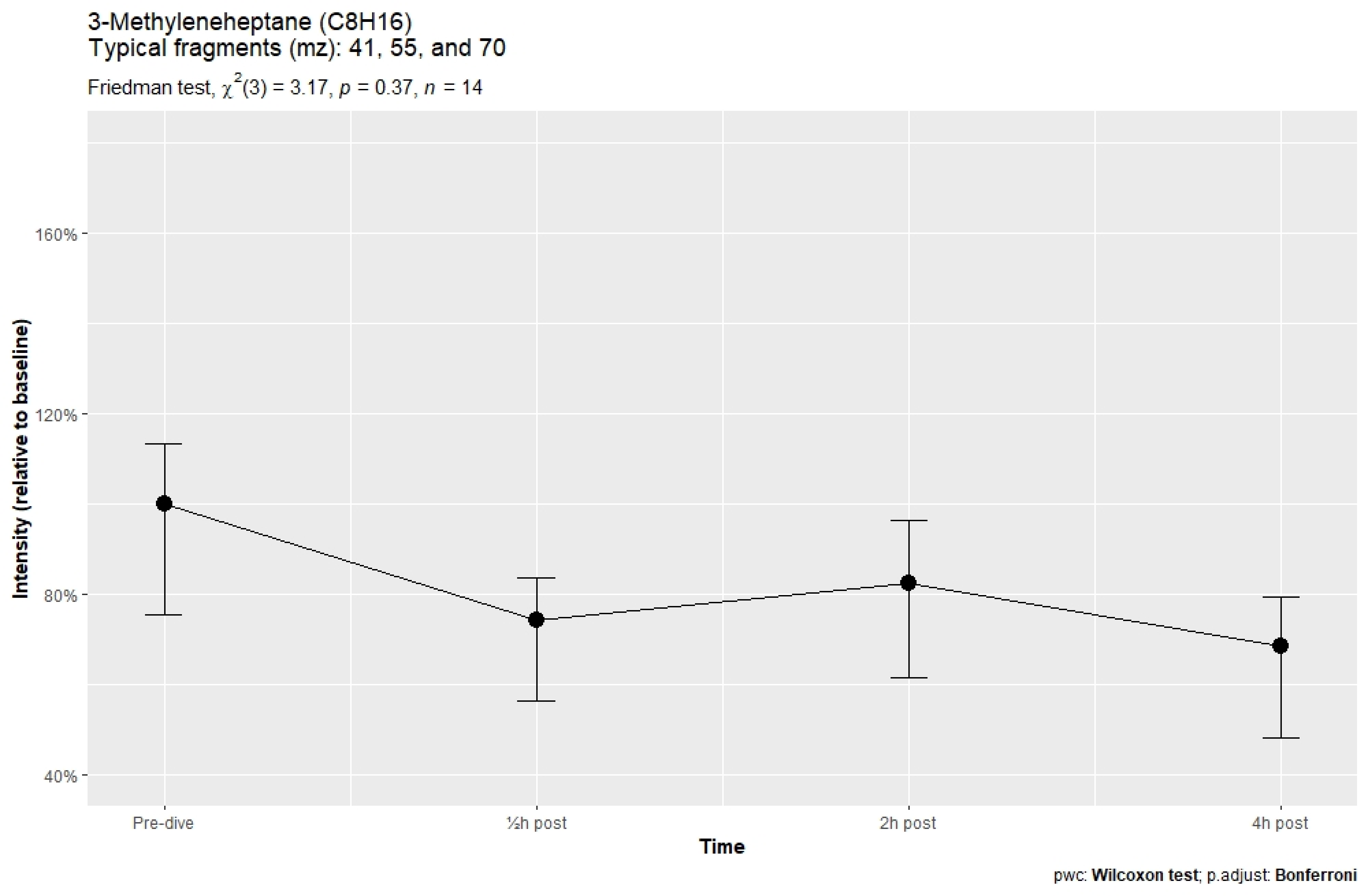

3-Methyleneheptane (C8H16)
Typical fragments (mz): 41, 55, and 70
Friedman test, χ2(3) = 3.17, p = 0.37, n = 14
160%
120%
80%
40%
Intensity (relative to baseline)
Pre-dive
½h post
2h post
4h post
Time
pwc: Wilcoxon test; p.adjust: Bonferroni

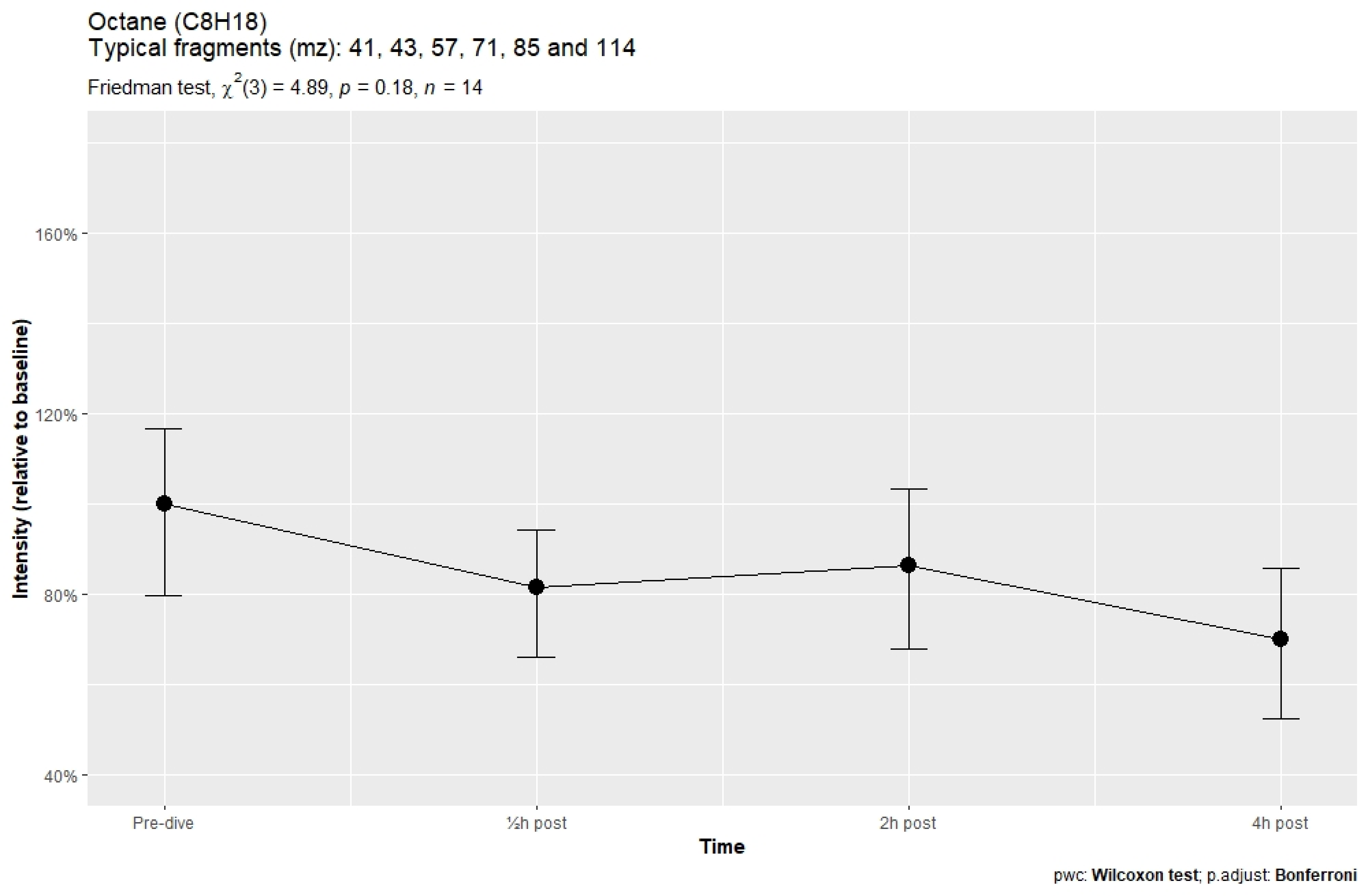

Octane (C8H18)
Typical fragments (mz): 41, 43, 57, 71, 85 and 114
Friedman test, χ2(3) = 4.89, p = 0.18, n = 14
160%
120%
80%
40%
Intensity (relative to baseline)
Pre-dive
½h post
2h post
4h post
Time
pwc: Wilcoxon test; p.adjust: Bonferroni

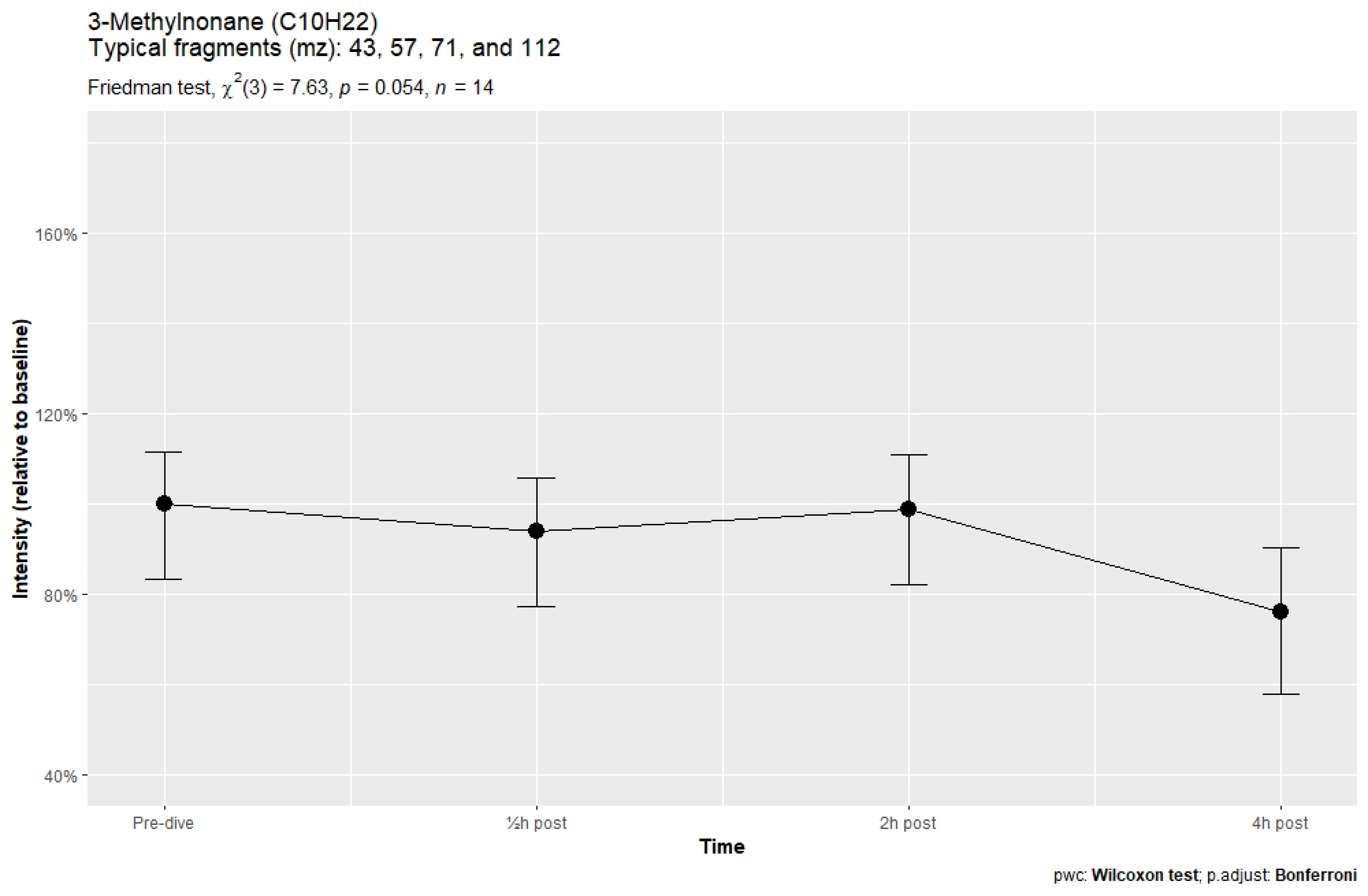
3-Methylnonane (C10H22)
Typical fragments (mz): 43, 57, 71, and 112
Friedman test, χ2(3) = 7.63, p = 0.054, n = 14
160%
120%
80%
40%
Intensity (relative to baseline)
Pre-dive
½h post
2h post
4h post
Time
pwc: Wilcoxon test; p.adjust: Bonferroni

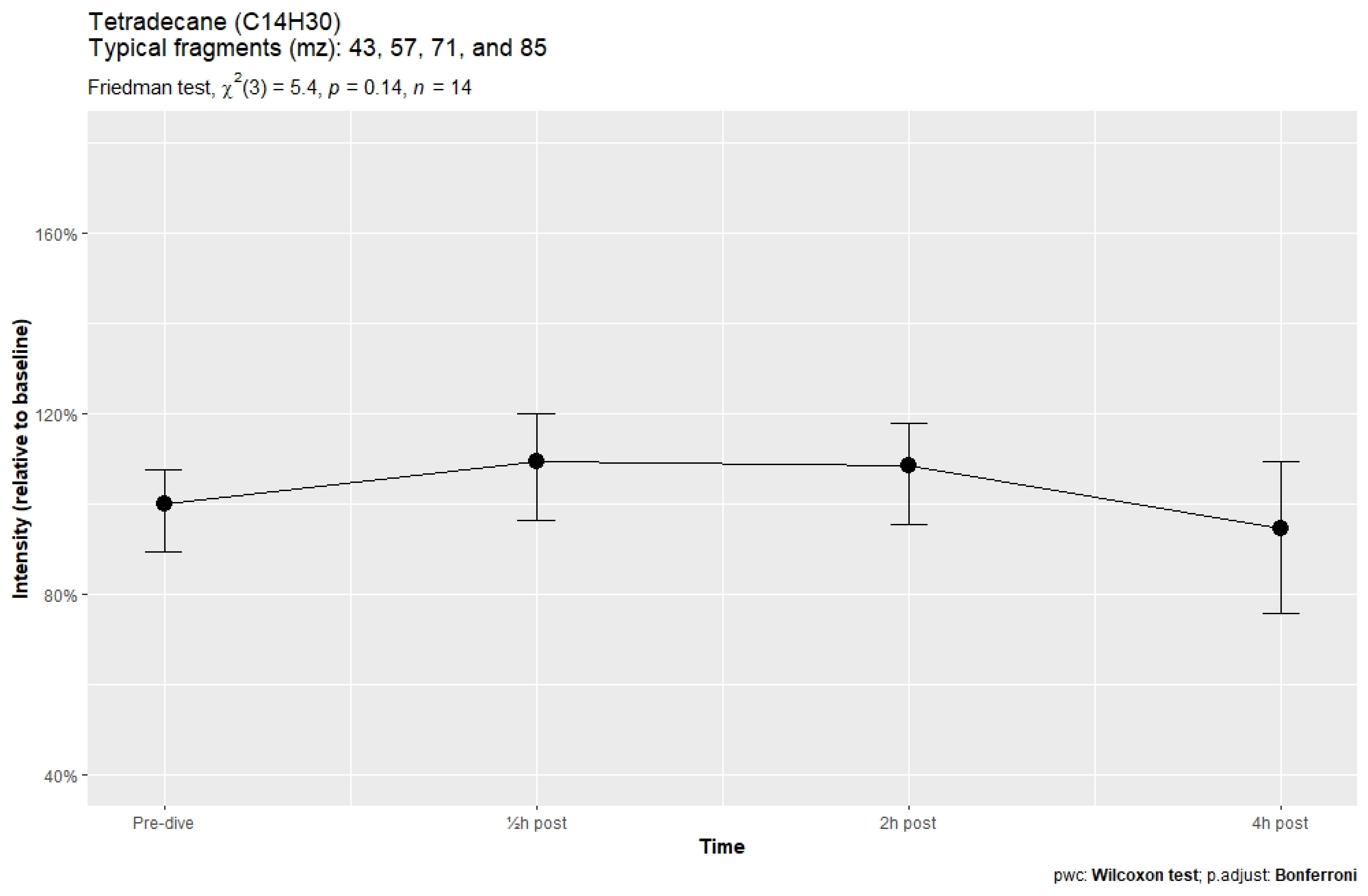
Tetradecane (C14H30)
Typical fragments (mz): 43, 57, 71, and 85
Friedman test, χ2(3) = 5.4, p = 0.14, n = 14
Intensity (relative to baseline)
160%
120%
80%
40%
Pre-dive
½h post
2h post
4h post
Time
pwc: Wilcoxon test; p.adjust: Bonferroni
