# Supplementary figures and images for: Pulmonary Oxygen Toxicity Through Exhaled Breath Markers After Hyperbaric Oxygen Treatment Table 6
Source: Front Physiol. 2022 May 10;13:899568. doi: 10.3389/fphys.2022.899568 (PMC9127798; doi:10.3389/fphys.2022.899568)

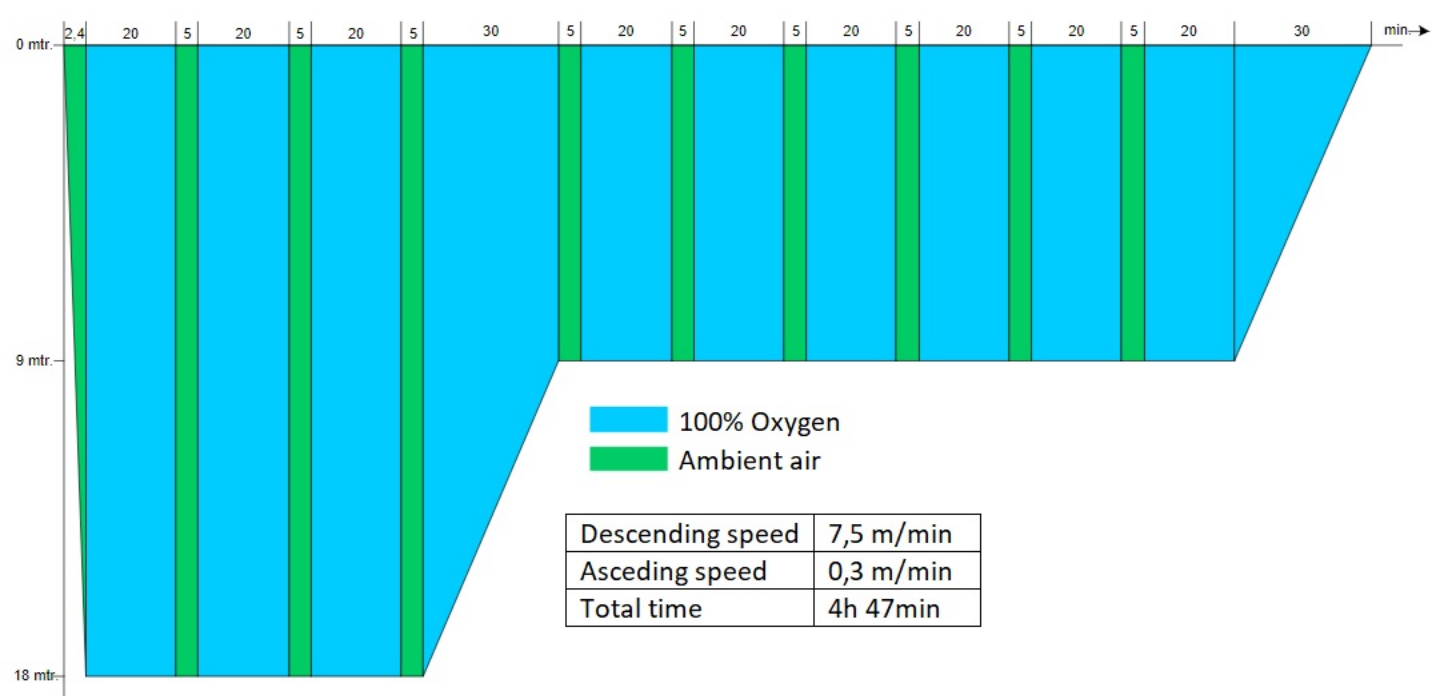

Supplement: Supplementary file 2 [file DataSheet1.pdf]
